# Bone sialoprotein stimulates cancer cell adhesion through the RGD motif and the αvβ3 and αvβ5 integrin receptors

**DOI:** 10.3892/ol.2024.14675

**Published:** 2024-09-09

**Authors:** Valentina Kottmann, Elena Kolpeja, Greta Baumkötter, Franziska Clauder, Ansgar Bokel, Franz Paul Armbruster, Philipp Drees, Erol Gercek, Ulrike Ritz

**Affiliations:** 1 Department of Orthopaedics and Traumatology, University Medical Center of The Johannes Gutenberg University Mainz, D-55131 Mainz, Germany; 2 Immundiagnostik AG, D-64625 Bensheim, Germany

**Keywords:** BSP, cell adhesion, integrin receptors, RGD, metastasis

## Abstract

Being implicated in bone metastasis development, bone sialoprotein (BSP) expression is upregulated in patients with cancer. While BSP regulates cancer cell adhesion to the extracellular matrix, to the best of our knowledge, the specific adhesive molecular interactions in metastatic bone disease remain unclear. The present study aimed to improve the understanding of the arginine-glycine-aspartic acid (RGD) sequence of BSP and the integrin receptors αvβ3 and αvβ5 in BSP-mediated cancer cell adhesion. Human breast cancer (MDA-MB-231), prostate cancer (PC-3) and non-small cell lung cancer (NSCLC; NCI-H460) cell lines were cultured on BSP-coated plates. Adhesion assays with varying BSP concentrations were performed to evaluate the effect of exogenous glycine-arginine-glycine-aspartic acid-serine-proline (GRGDSP) peptide and anti-integrin antibodies on the attachment of cancer cells to BSP. Cell attachment was assessed using the alamarBlue^®^ assay. The present results indicated that BSP supported the adhesion of cancer cells. The RGD counterpart GRGDSP peptide reduced the attachment of all tested cancer cell lines to BSP by ≤98.4%. Experiments with anti-integrin antibodies demonstrated differences among integrin receptors and cancer cell types. The αvβ5 antibody decreased NSCLC cell adhesion to BSP by 84.3%, while the αvβ3 antibody decreased adhesion by 14%. The αvβ3 antibody decreased PC-3 cell adhesion to BSP by 46.4%, while the αvβ5 antibody decreased adhesion by 9.5%. Adhesion of MDA-MB-231 cells to BSP was inhibited by 54.7% with αvβ5 antibody. The present results demonstrated that BSP-induced cancer cell adhesion occurs through the binding of the RGD sequence of BSP to the cell integrin receptors αvβ3 and αvβ5. Differences between cancer types were found regarding the mediation via αvβ3 or αvβ5 receptors. The present findings may explain why certain cancer cells preferentially spread to the bone tissue, suggesting that targeting the RGD-integrin binding interaction could be a promising novel cancer treatment option.

## Introduction

Accounting for ~10 million cancer deaths recorded in 2020, cancer is a major cause of death worldwide (1). Among these patients, ~90% die from secondary cancer rather than primary cancer (2). Cancer most frequently metastasises to the lymph nodes, lungs, liver and bones (3,4). Secondary bone cancers are commonly observed in advanced stages of breast (76–100%) and prostate cancer (76–100%), as well as in lung (26–50%) and kidney cancer (26–50%) (5). Skeletal metastases affect the bone remodelling process by stimulating bone formation (osteoblastic/osteosclerotic phenotype), enhancing bone resorption (osteolytic phenotype) or increasing both bone synthesis and degradation (mixed phenotype). For instance, the majority of skeletal metastases in patients with prostate cancer are considered osteosclerotic (6,7), whereas most breast cancer bone metastases are osteolytic (8). Osseous metastases can have a serious impact on the skeleton, ultimately affecting the patient's quality of life (QOL) (9). Once metastatic bone disease has formed, the median overall survival is 6 months (9). However, the life expectancy of a patient can range from 2.8 to 57 months, depending on the primary cancer (9,10). In most cases, there is no cure for bone metastasis, but the progression of the disease can be delayed. Therefore, improving the understanding of the metastatic cascade may help to advance treatment strategies and improve the QOL of patients.

Bone sialoprotein (BSP) is part of the small integrin-binding ligand N-linked glycoprotein (SIBLING) family of proteins (11). It is an acidic, glycosylated phosphoprotein almost exclusively expressed in mineralised tissues (12). Bone modelling and remodelling is mediated by BSP (13) and BSP is secreted into the non-mineralised extracellular matrix (ECM) when the osteoid is formed and the bone tissue is mineralised (14,15). To date, several functions of BSP have been described, such as the binding and potential induction of hydroxyapatite nucleation (16,17), and the stimulation of osteoclast differentiation (18). A previous review has described the role of BSP in bone development and turnover in detail (13). However, BSP is also involved in cancerous growth and metastasis. Cancer cell proliferation (19), migration (20), invasion (21), tumour cell evasion of immune system surveillance (22) and angiogenesis (23) are all stimulated by BSP. This strongly indicates that BSP plays a crucial role in driving cancer progression. BSP also regulates cancer cell adhesion (24,25) and the adhesion of cancer cells to BSP appears to be mediated by the binding of the arginine-glycine-aspartic acid (RGD) sequence of BSP to cell surface integrin receptors (26).

Integrins are a large family of cell adhesion molecules (to date the 18 α- and eight β-integrin subunits have been described in humans) and they recognise various integrin ligands, for example, fibronectin (FN), vitronectin (VN) and BSP (27,28). Integrin αvβ3 (VN receptor) on osteoclasts, osteosarcoma and osteoblast-like cells can bind to the RGD motif of BSP to induce cell attachment (28,29). This sequence is highly conserved among the SIBLING protein family (11), indicating an essential regulatory function. The RGD cell attachment domain of BSP is located at the C-terminus (30) and is flanked by tyrosine residues, which can be post-translationally modified, for instance by sulfation (31). The C-terminal tyrosine-rich region in BSP may drive integrin-mediated cell attachment, independent of the RGD sequence (29,31). Glycosylation modification (N-linked and O-linked glycosylation) of BSP potentially alters the activity of the protein as well as inhibits cell attachment (32). BSP can also bind to glycosaminoglycans, such as heparin, in an ionic-dependent manner, to increase the cell-binding activity of the RGD motif to the transmembrane integrin receptors (33). The heparin-binding sequence of BSP itself potentially requires the presence of the RGD domain to sufficiently induce cell adhesion (34).

The RGD motif of BSP was reported to bind to αvβ5 on human breast cancer cells (SKBR3) to stimulate cell attachment (35). At present, it is still not clearly defined which integrin receptors are involved in BSP-mediated cancer cell attachment, as findings from different studies seem contradictory. Sung *et al* (26) reported that the adhesion of the human melanoma cancer cell line MDA-MB-435 to BSP was αvβ5-dependent, whereas Byzova *et al* (35) showed that BSP-adhesion of MDA-MB-435 was αvβ3-mediated. In addition, MDA-MB-435 cells moderately to strongly express αvβ3 and αvβ5 integrin receptors (26). Furthermore, whether BSP can bind to other RGD-binding integrins on cancer cells, such as αvβ6, to induce cell adhesion remains unclear. Cells may also employ different integrin receptors in adhesion and chemotaxis (26). In addition, most studies have focused on osteosarcoma, breast cancer or melanoma, whereas little is known about the relation between BSP and the adhesion of prostate and lung cancer cells. The human prostate adenocarcinoma cell line PC-3 and the human lung cancer cell line NCI-H460 express both αvβ3 and αvβ5 integrin receptors, although the expression levels vary from weak to very strong (36–40).

Integrins promote tumour progression and metastasis by regulating tumour cell survival, proliferation, migration and invasion (41). Expression of the integrins αvβ3 and αvβ5 in tumour cells is associated with cancer progression (41). Interestingly, elevated BSP expression in the sera and in tumour sections of cancer patients is correlated with disease progression and poor survival as well (42–44). Furthermore, BSP has been identified as a potential risk factor for bone metastasis development in breast and lung cancer patients (45,46). Given that αvβ3 and BSP are co-expressed in prostate cancer cell lines (47) and primary breast invasive ductal carcinoma samples (21), the binding of BSP to integrins may contribute to tumour progression by connecting cancer cells to the ECM and activating downstream signalling pathways.

Signalling pathways that are stimulated upon BSP-integrin binding or BSP treatment include, for instance, focal adhesion kinase (FAK), extracellular signal-regulated kinase (ERK), phosphatidylinositol 3-kinase (PI3K)/protein kinase B (AKT) and AP-1 signalling (20,25). These signalling pathways promote tumour growth (48) and metastasis (49,50). BSP can not only bind to integrins but can also bind to and activate pro matrix metalloproteinase (MMP)-2 (51). MMPs play an important role in tumour metastasis by degrading the ECM and basement membrane to promote cancer cell migration and invasion (52). Karadag *et al* (53) demonstrated enhanced cancer cell invasiveness when BSP formed a trimolecular complex with αvβ3 and matrix metalloproteinase (MMP)-2. Cancer cell invasion was RGD-dependent as the replacement of the RGD motif of BSP with the motif KAE (lysine-alanine-glutamic acid) inhibited cancer cell invasiveness (53). This highlights the importance of the RGD sequence in cancer cell chemotaxis.

Collectively, the current literature suggests that the interactions of BSP with αvβ3-integrins play a central role in driving cancer cell chemotaxis and promoting the development of skeletal metastases in malignant cells. BSP-integrin-induced cancer cell adhesion and motility seem to be RGD-dependent. However, the role of BSP/αvβ5-integrin binding in cancer cell adhesion and tumour progression remains unclear. Because the BSP-integrin interactions could be essential for the progression of cancer and metastasis, the present study aimed to elucidate whether BSP enhances the adhesion of cancer cells by binding its RGD sequence to the integrin receptors αvβ3 and αvβ5. Adhesion of breast, prostate and lung cancer cell lines was investigated using a cell adhesion assay. We hypothesised that breast adenocarcinoma, prostate adenocarcinoma and large-cell lung cancer cells adhere to BSP and that this attachment of cancer cells to BSP is mediated by the RGD sequence of BSP and either the αvβ3-integrin receptors and/or the αvβ5-integrin receptors.

## Materials and methods

### Reagents and antibodies

Human plasma FN was purchased from Merck Millipore. Human BSP (huBSP2) was provided by Immundiagnostik AG. Human VN was purchased from Sartorius (Göttingen, Germany). Phosphate-buffered saline (PBS) was purchased from Sigma-Aldrich Chemie GmbH (Taufkirchen, Germany). FN-derived RGD peptide glycine-arginine-glycine-aspartic acid-serine-proline (GRGDSP) was also purchased from Sigma-Aldrich (Merck KGaA). Isotype control, anti-mouse IgG1 (catalogue no. 02-6100) was purchased from Gibco (Thermo Fisher Scientific, Inc.). Anti-integrin αvβ3 mouse monoclonal antibody, (clone LM609; catalogue no. MAB1976Z) and anti-integrin αvβ5 mouse monoclonal antibody (clone P1F6; catalogue no. MAB1961Z) were obtained from Millipore (Merck KGaA). AlamarBlue™ cell viability reagent (catalogue no. DAL1100) was purchased from Invitrogen (Thermo Fisher Scientific, Inc.).

### Cell culture

The human breast carcinoma cell line MDA-MB-231 [DSMZ ACC 732 (RRID:CVCL 0062)] was purchased from Leibniz Institute DSMZ-German Collection of Microorganisms and Cell Cultures. Established in 1972, this cell line originated from the pleural effusion of a woman who had undergone chemotherapy for breast cancer. MDA-MB-231 cells were cultured in Roswell Park Memorial Institute (RPMI) 1640 medium (Life Technologies, Ltd.), supplemented with 10% heat-inactivated fetal calf serum (FCS; Biochrom GmbH), 1% penicillin-streptomycin (P/S; Sigma-Aldrich; Merck KGaA), 2 mM L-Alanyl-L-Glutamine (Sigma-Aldrich; Merck KGaA), 5 ml Minimum Essential Medium non-essential amino acids (MEM NEAA), 100X (Gibco; Thermo Fisher Scientific, Inc.) and 1 mM sodium pyruvate (Gibco; Thermo Fisher Scientific, Inc.). The human prostate cancer cell line PC-3 (RRID:CVCL 0035) was kindly provided by Dr. Eva Jüngel and was grown in Iscove's Basal Medium (Biochrom GmbH), supplemented with 10% heat-inactivated FCS and 1% P/S. The human large-cell lung carcinoma cell line NCI-H460 [H460] [ATCC HTB-177 (RRID:CVCL 0459)] was grown in RPMI 1640 medium, supplemented with 10% heat-inactivated FCS and 1% P/S. Authentication of cells was conducted using short tandem repeat profiling. Cells were confirmed to be mycoplasma-free. All cell lines were grown in a humidified environment at 37°C and 5% CO_2_. Medium was renewed twice a week.

### Cell adhesion assay

The adhesion assay protocol by Oliveira-Ferrer *et al* (54) was modified and used in the present study, and the adhesion assay was carried out once in quintuples. Briefly, 24-well suspension culture plates (Cellstar^®^; Greiner Bio-One GmbH) were pre-coated (250 µl/well) with PBS, 10 µg/ml human plasma FN and 3 µg/ml huBSP2 diluted in PBS. The concentration of BSP was selected based on preliminary, unpublished data from the authors' laboratory. The plates were incubated overnight at room temperature. Cultured cell lines were washed with PBS, detached from the flask using Accutase (Sigma-Aldrich; Merck KGaA), centrifuged (5 min at 353 × g and room temperature) and diluted to 2.4×10^5^ cells/ml in reduced growth medium (GM) with 2.5% FCS. After removing the supernatant from the pre-coated suspension culture plates, cells were added to the wells (6×10^4^ cells/250 µl/well) and were allowed to adhere for 2 h at 37°C and 5% CO_2_. The supernatant containing the unattached cells was then removed and wells were washed once with PBS (500 µl/well). AlamarBlue™ cell viability reagent was diluted 1:10 in reduced GM according to the manufacturer's instructions and added to the plate (500 µl/well). Blank wells with no coating solution and no cells were also included. Fluorescence was read at 540 nm excitation wavelength and 590 nm emission wavelength with a fluorescence multi-well reader (GloMax^®^ Multi+ Detection System; Promega Corporation) after 4 h (37°C, 5% CO_2_) using the alamarBlue^®^ assay.

The alamarBlue^®^ assay is a common method to study cell viability and cytotoxicity (55). AlamarBlue™ cell viability reagent (catalogue no. DAL1100) contains resazurin and upon accepting electrons, the initial blue-coloured and non-fluorescent resazurin-based solution changes to the pink-coloured and fluorescent resorufin solution (56). The level of fluorescence is directly proportional to the number of viable cells.

The alamarBlue^®^ assay was carried out in quadruplicate (100 µl/well) on a 96-well microplate (Greiner Bio-One GmbH) to measure cell viability (metabolic activity). Cell viability was used to quantify the number of adherent cells.

### Dose-response curve of BSP and adherent cells

A BSP dose-response curve (concentration-dependent adhesion assay) was established for each cancer cell line. In short, huBSP2 was serially diluted to various concentrations (0 to ≤10 µg/ml) in PBS and coated in triplicate (250 µl/well) on 24-well suspension culture plates overnight at room temperature. The ensuing protocol steps were identical to the previous experiment.

The BSP concentration-dependent adhesion assay was carried out three times in total and the results were pooled following completion of the experiments. The concentration of BSP needed to induce 80% of maximal cell adhesion (EC_80_) was calculated for each cell line. In short, EC_80_ values were obtained by plotting the dose response data (BSP concentrations against mean fluorescence readings) using a sigmoidal curve with a variable slope. The EC_80_ of huBSP2 was used in the subsequent RGD-binding and integrin-binding assays.

### RGD-binding assay

To investigate the mechanistic link behind the cell adhesion of human cancer cell lines to BSP and hence the ECM, an RGD-binding assay was performed by Stachurska *et al* (57) with the following modification. 24-well suspension culture plates were pre-coated with 10 µg/ml human VN in sextuplicate (250 µl/well) and the EC_80_ of huBSP2 (15–18 wells, 250 µl/well). The EC_80_ of huBSP2 was derived from the dose-response curve of BSP and adherent cells. Plates were incubated overnight at room temperature. The next day, cultured cells were diluted to 2.4×10^5^ cells/ml in reduced GM with 2.5% FCS and cell suspensions were incubated for 30 min (37°C and 5% CO_2_) with FN-derived RGD peptide GRGDSP at various concentrations (0 to ≤120 µM). In the intervening time, the supernatant of the plate coating solution was removed and the 24-well plate was incubated with 3% bovine serum albumin (BSA; PAA Laboratories GmbH) in PBS (250 µl/well) for 30 min at RT to block non-specific binding sites. After removal of the blocking solution, wells were washed twice with PBS (500 µl/well) and the incubated cell suspension was added to the plate (6×10^4^ cells/250 µl/well). Following a 2-h incubation period (37°C, 5% CO_2_), the supernatant was discarded and wells were washed again with PBS (500 µl/well). AlamarBlue™ cell viability reagent (1:10 in reduced GM) was added to the wells (500 µl/well). A blank was also pipetted. The fluorescence signal was read after 4 h (37%, 5% CO_2_) to measure cell viability. Adherent cells were also imaged with phase-contrast microscopy to evaluate the degree of cell spreading. The RGD-binding assay was performed in triplicate in three independent experiments.

### Integrin-binding assay

The contribution of the cell surface integrin receptors αvβ3 and αvβ5 in the cell adhesive activities of BSP was determined by performing a receptor inhibition assay. The experiment was carried out as described in the RGD-binding assay with some modifications. 10 µg/ml human VN and the EC_80_ of huBSP2 were coated onto a 24-well suspension culture plate at 250 µl per well, in sextuplicate each. Following incubation overnight at room temperature, the supernatant of the coated plate was removed and replaced with 3% BSA blocking solution (250 µl/well) for 30 min at room temperature. Meanwhile, cell suspensions of cultured cell lines (2.4×10^5^ cells/ml in reduced GM with 2.5% FCS) were incubated with 10 µg/ml anti-integrin αvβ3 mouse monoclonal antibody, 10 µg/ml anti-integrin αvβ5 mouse monoclonal antibody or 10 µg/ml isotype control in duplicate (250 µl/well) for 60 min (37°C, 5% CO_2_). The blocking solution was removed and the plate was washed twice with PBS (500 µl/well). Cells were then added to the coated culture plate (6×10^4^ cells/250 µl/well) and incubated for 2 h (37°C, 5% CO_2_). The supernatant containing the non-adherent cells was discarded and the plate was washed once with PBS (500 µl/well). PBS was removed through vacuum suction and 500 µl of alamarBlue™ cell viability reagent (1:10 in 2.5% FCS GM) was added into each well. A blank was also included. The attached cells were quantified by measuring the cell viability after 4 h (37°C, 5% CO_2_) using the alamarBlue™ assay. Two independent integrin-binding experiments were carried out in duplicate.

### Statistical analysis

The cell viability assay was used as an indirect measurement of cell adhesion. The fluorescence signals were corrected by subtracting the mean fluorescence value of the blank (i.e. auto-fluorescence of alamarBlue™ cell viability reagent) from all individual sample readings. The individual sample readings of each group were averaged for quantitative analysis of cell adhesion. Percentages were calculated relative to the negative control as follows: % control=[(control-treated)/control] ×100.

Values are presented as mean ± standard deviation. One-way ANOVA, followed by Dunnett's multiple comparison test where appropriate, was performed using GraphPad Prism 9 software for Windows (GraphPad Software, Inc.; Dotmatics). P<0.05 was considered to indicate a statistically significant difference.

## Results

### Cancer cells adhere to BSP in vitro

**Figure 1.**
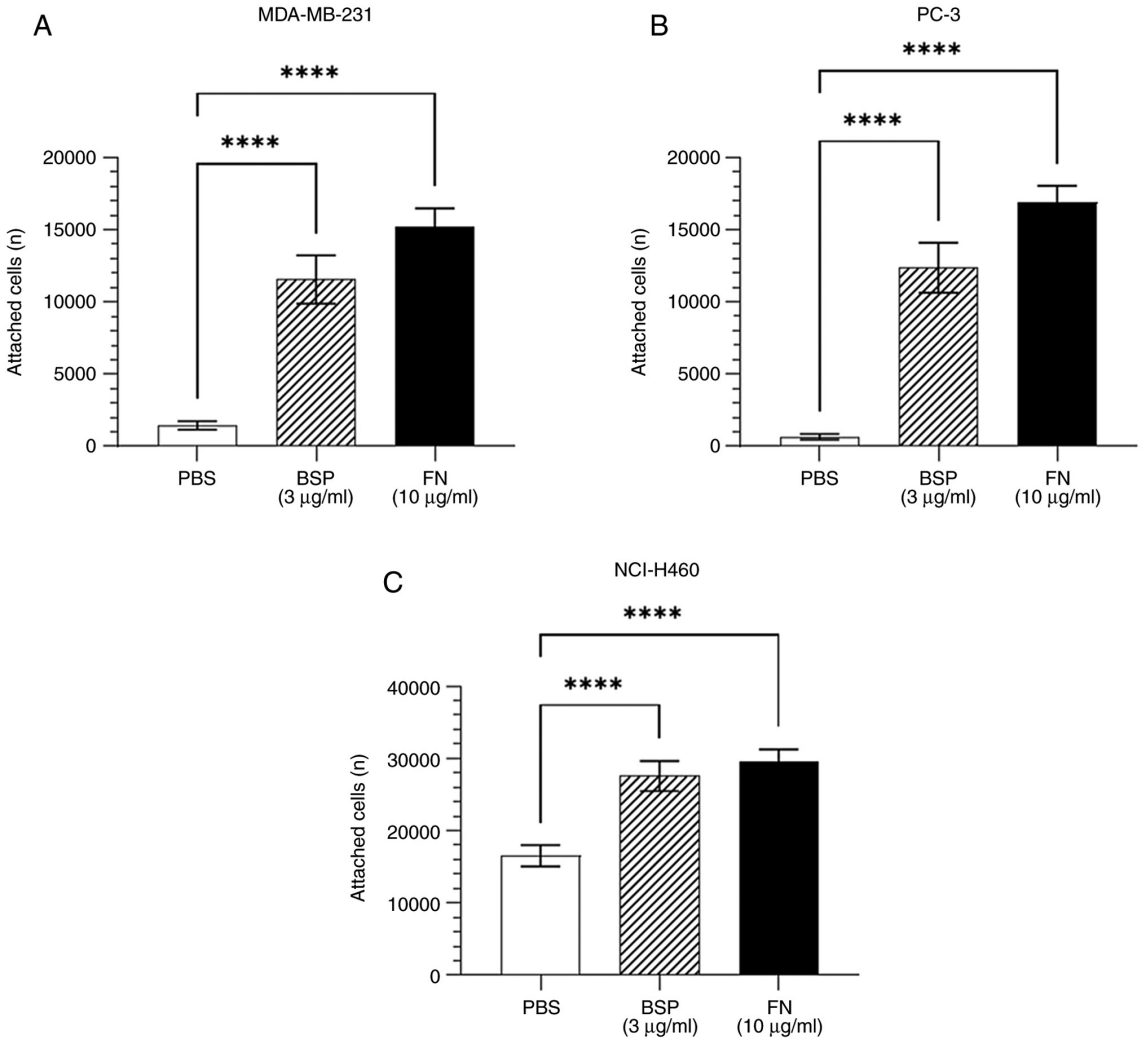
Adhesion of cancer cell lines to various surface coatings, as determined by the alamarBlue^®^ assay. (A) MDA-MB-231 (n=5), (B) PC-3 (n=5) and (C) NCI-H460 (n=5) cancer cells attached to PBS, BSP and FN. Error bars represent the standard deviation. ****P<0.0001 vs. negative control (PBS). PBS, phosphate-buffered saline; BSP, bone sialoprotein; FN, fibronectin.

Adhesion assays were performed to determine whether human cancer cells adhere to BSP. As shown in Fig. 1, BSP significantly supported the attachment of breast adenocarcinoma MDA-MB-231, prostate adenocarcinoma PC-3 and large-cell lung cancer NCI-H460 cell lines compared with the negative control (PBS).

### Cancer cell adhesion to BSP is dose-dependent

**Figure 2.**
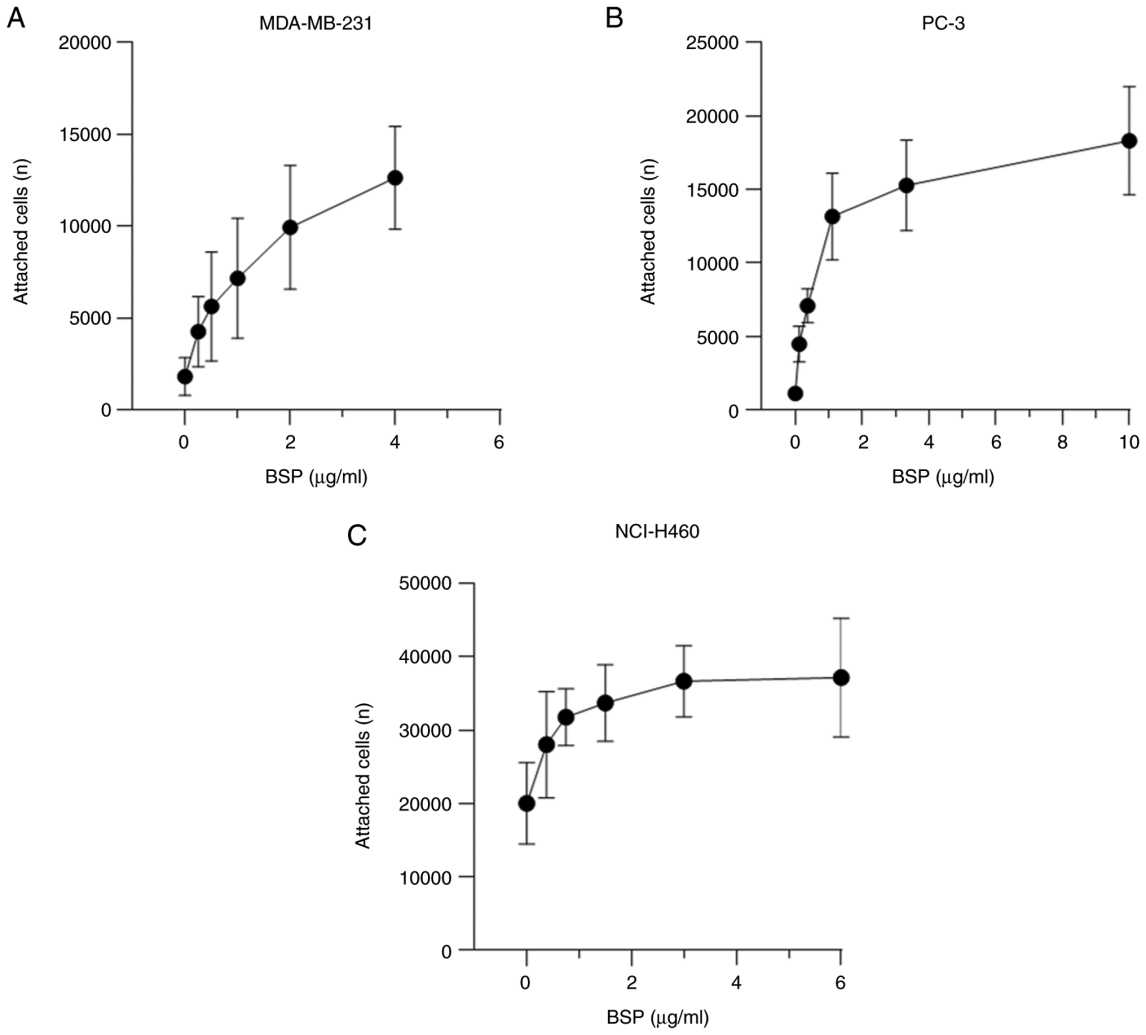
Increased attachment of human cancer cells to BSP with increasing protein concentrations, as determined by the alamarBlue^®^ assay. (A) MDA-MB-231 (n=9), (B) PC-3 (n=9) and (C) NCI-H460 (n=9) cancer cells attached to BSP after 2 h. Error bars represent the standard deviation. BSP, bone sialoprotein.

The current findings demonstrate a dose-dependent stimulation of cell attachment to BSP (Fig. 2). The dose-response curve of BSP and adherent breast adenocarcinoma MDA-MB-231 cells was constructed using the following huBSP2 concentrations: 4, 2, 1, 0.5, 0.25 and 0 µg/ml. huBSP2 was serially diluted to 10, 3.33, 1.11, 0.37, 0.123 and 0 µg/ml for PC-3, while it was serially diluted to 6, 3, 1.5, 0.75, 0.375 and 0 µg/ml for NCI-H460. The amount of adherent cancer cells increased with increasing protein concentrations. The concentration of BSP that led to EC_80_ was 1.209 µg/ml for MDA-MB-231, 3.5 µg/ml for PC-3 and 1.403 µg/ml for NCI-H460.

### RGD sequence strongly regulates the adhesion of cancer cells to BSP

**Figure 3.**
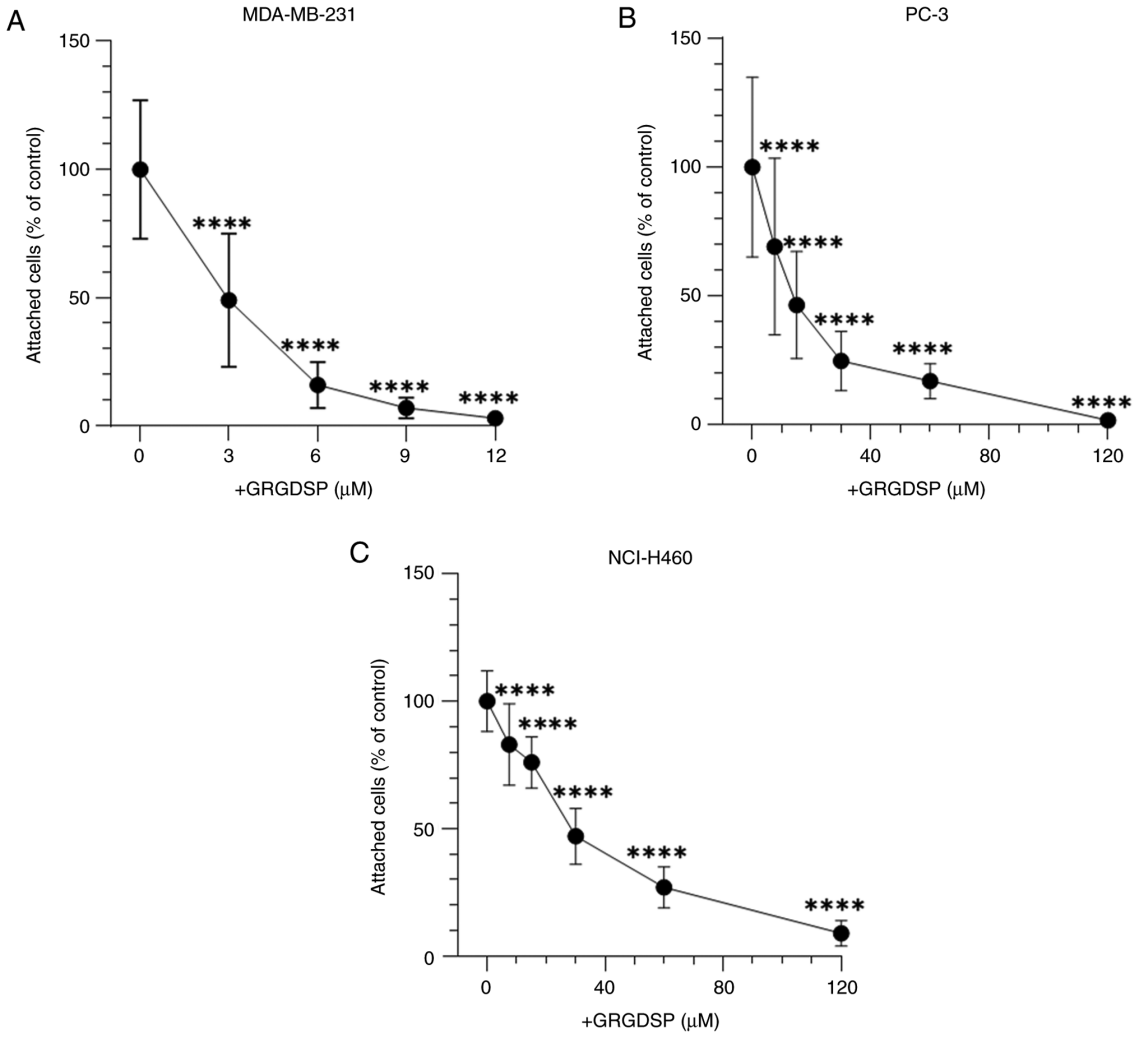
Effects of exogenous GRGDSP peptide on cancer cell adhesion to BSP, as determined by the alamarBlue^®^ assay. (A) MDA-MB-231 (n=9), (B) PC-3 (n=9) and (C) NCI-H460 (n=9) cells were incubated for 30 min at 37°C in the presence of increasing concentrations of GRGDSP peptide. Afterwards, cells were cultured for 2 h on BSP-coated plates and attached cells were examined. Data are reported as percentages compared with 0 µM GRGDSP (100%). Error bars represent the standard deviation. ****P<0.0001 vs. negative control (0 µM GRGDSP). GRGDSP, glycine-arginine-glycine-aspartic acid-serine-proline; BSP, bone sialoprotein.

**Figure 4.**
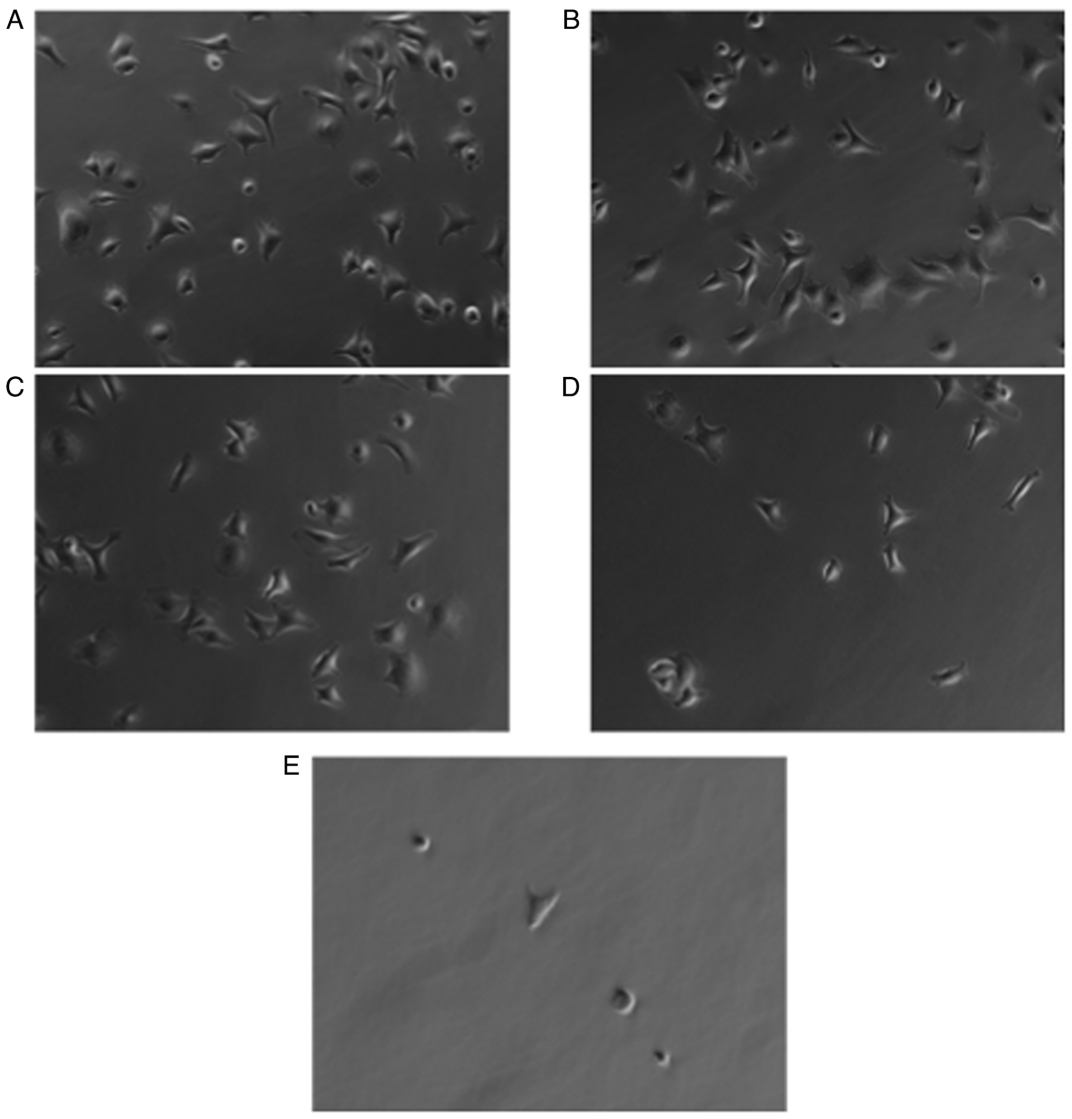
Effect of incubation with (A) 0, (B) 7.5, (C) 15, (D) 30 or (E) 60 µM glycine-arginine-glycine-aspartic acid-serine-proline peptide for 30 min and 2 h culturing on BSP-coated plates on the adhesion to BSP and morphology of PC-3 cells (magnification, ×20). BSP, bone sialoprotein.

The involvement of the RGD sequence in BSP-mediated cancer cell adhesion was examined. GRGDSP significantly inhibited the attachment of all cancer cell lines to BSP in a concentration-dependent manner (Fig. 3). Cell shape of cancer cells was elongated in the absence and at low concentrations of GRGDSP peptide (Fig. 4A-C). With elevated levels of RGD-containing peptide, cells became more round-shaped and detached from BSP-coated plates (Fig. 4D and E).

The exogenously added RGD peptide had a limited effect on the level of breast adenocarcinoma MDA-MB-231 and large-cell lung cancer cell NCI-H460 adhesion to VN (data not shown). Cell adhesion to VN decreased by 7.6% for MDA-MB-231 at 12 µM RGD-containing peptide compared with the prior incubation with 0 µM GRGDSP [55723±5560 (fluorescence 560/590 nm) vs. 60311±8150] and by 6.3% for NCI-H460 at 120 µM RGD motif-containing peptide (39549±5697 vs. 42217±6853). Exogenous RGD peptide could inhibit prostate carcinoma cell attachment, though the inhibition achieved with GRGDSP was not complete (data not shown). 120 µM GRGDSP strongly inhibited the attachment of PC-3 to VN by 62.8% with respect to 0 µM GRGDSP (13226±7038 vs. 35575±14194).

### Binding of cancer cells to BSP is integrin-mediated

**Figure 5.**
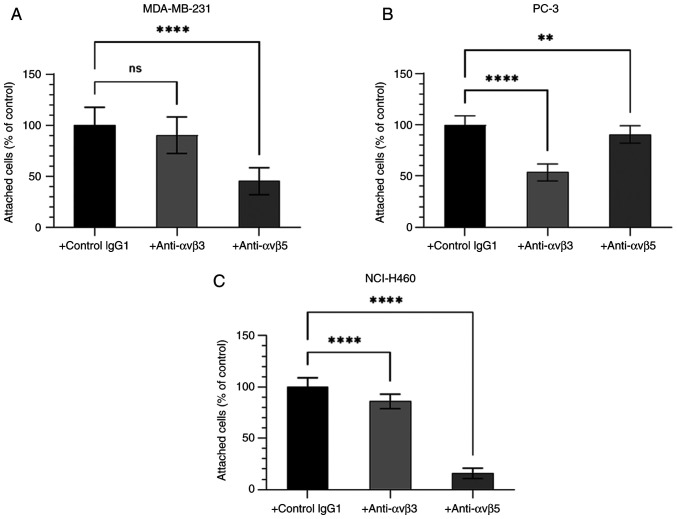
Effect of anti-αvβ3 or anti-αvβ5 antibody on cancer cell adhesion to BSP, as determined by the alamarBlue^®^ assay. (A) MDA-MB-231 (n=4), (B) PC-3 (n=4) and (C) NCI-H460 (n=4) cells were incubated for 60 min at 37°C in the presence of 10 µg/ml isotype control IgG1, 10 µg/ml anti-αvβ3 antibody or 10 µg/ml anti-αvβ5 antibody. Afterwards, cells were cultured for 2 h on BSP-coated plates and attached cells were examined. Data are reported as percentages compared with 10 µg/ml isotype control IgG1 (100%). Error bars represent the standard deviation. **P<0.01; ****P<0.0001 vs. negative control IgG1. BSP, bone sialoprotein; ns, not significant.

Since the RGD domain can bind to integrin receptors, the function of the integrin receptors αvβ3 and αvβ5 in cancer cell adhesion to BSP was further characterised. The adhesion of human cancer cell lines MDA-MB-231, PC-3 and NCI-H460 to BSP was dependent upon the αvβ3-integrin receptors and the αvβ5-integrin receptors (Fig. 5). The degree of contribution of integrins αvβ3 and αvβ5 to BSP-induced cancer cell adhesion differed among cell lines.

## Discussion

Several studies have shown that BSP is involved in multiple steps in tumour progression, including cancer cell attachment (35). Cancer cell adhesion to the extracellular matrix of bone is a critical step in the metastatic cascade. Previous research suggests that the RGD sequence of BSP can bind to αvβ3 and αvβ5 integrins on cancer cells to induce cancer cell attachment (26,35). Elucidating the role of BSP in cancer cell attachment will help to understand the development of bone metastases, improve precision medicine and ultimately advance current cancer treatment strategies.

The present authors performed several *in vitro* adhesion assays to identify the mechanism behind the homing of human breast, prostate and lung cancer cells to the bone. We hypothesised that cancer cell lines adhere to BSP and that the adhesion is regulated by the RGD-binding domain of BSP and the αvβ3-integrin and/or αvβ5-integrin receptors. To the best of our knowledge, the present study is the first to demonstrate that large-cell lung carcinoma NCI-H460 cells adhere to BSP. The current results also showed that BSP promotes the cell attachment of prostate adenocarcinoma PC-3 and breast adenocarcinoma MDA-MB-231 cells, which is in agreement with previous studies (25,26). We did not investigate downstream signalling pathways in BSP-induced cancer cell adhesion. However, previous studies showed that BSP is able to stimulate several signalling pathways involved in cancerous growth and spread (20,25). Gordon *et al* (25) observed BSP-mediated activation of FAK-ERK after adherence of PC-3 and MDA-MB-231 cells to rat BSP for one hour. Importantly, an intact RGD-integrin binding sequence of BSP is required for FAK and MAPK activation, as MDA-MB-231 cells infected with mutated BSP (BSP-KAE) exhibit lower FAK and ERK phosphorylation (25). Similar to BSP-induced adhesion of PC-3 and MDA-MB-231 cells, adherence of NCI-H460 cells to BSP potentially activates RGD-dependent FAK and ERK signalling. The attachment of all three cancer cell lines to BSP was extensively mediated by the RGD motif. This was previously observed in murine cementoblasts (58), murine osteoblast-like cells (29), human bone fibroblasts (59) and human skin fibroblasts (59) as well. By contrast, van der Pluijm *et al* (60) reported no effect of adding commercially available (non-BSP derived) GRGDS peptide at concentrations up to 300 µM on the attachment of the human breast cancer cell line MDA-MB-231 to ECM of human bone cells. Nonetheless, cyclic synthetic BSP peptides containing the EPRGDNYR sequence were strong inhibitors of breast cancer cell adhesion to human ECM at concentrations of only 2 µM (60). The authors argued that the adhesion of MDA-MB-231 cells to the ECM of bone is not solely regulated by the RGD sequence of BSP. In the present study, FN-derived RGD-peptide GRGDSP was effective, almost completely blocking (97%) breast adenocarcinoma adhesion to BSP. Mintz *et al* (59) also reported that 0.4 mM synthetic GRGDS peptide completely inhibited the attachment of human bone cells and human skin fibroblasts to rat BSP. The contribution of tyrosine-rich repeat to BSP cell attachment activity was likely negligible as attachment of breast cancer, prostate adenocarcinoma and NSCLC cells was greatly diminished with GRGDSP (31). Cell attachment independent of tyrosine residues was previously described in osteopontin (OPN), another member of the SIBLING family of proteins, as well (61).

The present study showed for the first time that the adhesion of the PC-3 cell line to BSP is mainly αvβ3-dependent, whereas the adhesion of the NCI-H460 cell line to BSP is essentially αvβ5-mediated. BSP-induced breast cancer adhesion mainly used αvβ5 receptors. The data also suggests that large-cell lung carcinoma cells partially use the αvβ3 integrin receptors to regulate BSP-induced cell adhesion, whereas prostate cancer cells use, to a small, but significant degree, the αvβ5 integrin receptors to regulate BSP-induced cell adhesion. Colon adenocarcinoma cells also use the integrins αvβ3 and αvβ5 to adhere to OPN (61). Depending on the cancer cell line, the integrin heterodimers αvβ3 and αvβ5 may preferentially bind to BSP. NCI-H460 cell adhesion to BSP was greatly diminished in the presence of αvβ5-integrin antibody. However, neither the αvβ3- nor the αvβ5-integrin antibody was able to completely inhibit BSP-induced adhesion of the NSCLC, prostate adenocarcinoma and breast adenocarcinoma cells. Stimulation of cancer cells with an exogenous agonist, such as phorbol 12-myristate 13-acetate (PMA) or adenosine diphosphate (ADP), after antibody incubation, could potentially increase the binding of the integrin receptors to BSP (62,63). However, the results of the present study showed that integrin activation was not required for the adhesion of cancer cells to BSP. Future studies should pre-treat cells with anti-αvβ3 and anti-αvβ5 integrin antibodies, followed by stimulation with 200 nM or 200 ng/ml PMA or various concentrations of ADP (2–2,000 µM) to examine the effect on breast adenocarcinoma, prostate adenocarcinoma and large-cell lung cancer cell attachment to BSP. BSP may also adjust the expression of integrins in cancer cells to regulate cancer cell adhesion. BSP-infected MDA-MB-231 and PC-3 cells display elevated levels of the integrin subunits αv, β3 and β5, leading to greater focal adhesion formation in relation to non-infected cells or cells infected with mutated BSP (BSP-KAE) (25). The expression of BSP by a cancer cell may increase the cell's potential to metastasise. Future studies should treat or infect cells with BSP prior to cell seeding.

As mentioned earlier, cells use integrin receptors in adhesion and chemotaxis differently, which could provide another possible explanation as to why the adhesion of cells to BSP was not completely blocked by integrin antibodies. Sung *et al* (26) observed that the attachment of MDA-MB-231 cells to BSP was αvβ5-dependent, whereas the αvβ3-integrin receptors mediated the migration of MDA-MB-231 cells to BSP-derived RGD peptides. The results in the present study suggested that integrins αvβ3 and αvβ5 differentially mediate cell adhesion depending on the cell type in which they are expressed. Sung *et al* (26) did not examine additional integrin receptors in the blocking experiments. Likewise, the current study only looked at the αvβ3- and the αvβ5-integrin receptors and did not quantify the expression levels of these receptors on the cell surface. However, previous research indicated that the breast adenocarcinoma cell line MDA-MB-231 and the prostate adenocarcinoma cell line PC-3 strongly express αvβ5 integrin receptors (26,38,64). Less is known about αvβ5 integrin receptors in the NSCLC cell line NCI-H460. One study reported weak expression levels of αvβ5 in NCI-H460 cells (39), while αvβ3 integrin receptor expression varies within and between cancer cell lines, ranging from low to moderate expression in the breast cancer cell line MDA-MB-231 (26,64) and from moderate to very high expression in the cancer cell lines NCI-H460 and PC-3 (36–38,40). It is therefore plausible that prior incubation with anti-integrin αvβ5 antibody led to a greater reduction in the number of attached MDA-MB-231 cells to BSP compared with the αvβ3 antibody incubation. By contrast, the similar expression levels of integrin αvβ5 and αvβ3 in PC-3 cannot explain the findings of the present investigation. The results seen in NCI-H460 should be interpreted with caution given the limited knowledge of αvβ5 integrin expression. Whilst integrin αvβ3 is expressed at a significantly greater level on NCI-H460 in comparison with integrin αvβ5, integrin αvβ3 may not be the main integrin receptor driving NSCLC cell attachment to BSP. This would also support the notion of the differential employment of integrins in cell adhesion and chemotaxis. Flow cytometric evaluation of αvβ5 integrin receptor expression on NCI-H460 cells will improve the current understanding of integrin-mediated large-cell lung cancer adhesion to BSP.

Other RGD-binding integrin receptors could have been involved in the cell adhesion assays. The breast adenocarcinoma cell line MDA-MB-231 expresses, apart from the αvβ5 and αvβ3 integrin receptors, also the αvβ6 RGD-binding integrin receptor (64) and the RGD-binding integrin subunit β1 (65). The prostate cancer cell line PC-3 expresses multiple RGD-binding integrin receptors including the αvβ5, αvβ3, αvβ6, α5β1 and αvβ1 receptors (38). NCI-H460 cells express the RGD-recognising integrin receptor α5β1 and the RGD-binding integrin subunits α5, αv, β1 and β6 (66). Cell-specific integrin receptor expression may also explain why different RGD peptide concentrations were needed to strongly or completely inhibit the attachment of the three cancer cell lines to BSP. The GRGDSP peptide potentially bound to integrin receptors on MDA-MB-231 cells at a higher affinity in contrast to PC-3 and NCI-H460 cells (67,68). Further tests will elucidate whether other integrin receptors, such as α5β1, αvβ1 or αvβ6, mediate BSP-induced cancer cell adhesion. Among these, αvβ6 is a promising candidate as the receptor can recognise the RGD sequence of OPN (61).

Additional BSP sequences could have regulated BSP-induced cancer cell attachment. The present study did not investigate the role of the heparin-binding sequence of human BSP, leucine-histidine-arginine-arginine-valine-lysine-isoleucine (LHRRVKI), in cancer cell adhesion. Other studies demonstrated that the heparin-binding sequence of rat BSP, phenylalanine-histidine-arginine-arginine-isoleucine-lysine-alanine (FHRRIKA), plays a role in BSP-mediated cell adhesion. For instance, Rezania and Healy (34) showed that less rat osteoblast-like cells attach to homogenously coated FHRRIKA surfaces when compared with surfaces coated with both RGD and FHRRIKA. Furthermore, no focal contact formation by bone cells is observed on homogenous FHRRIKA-coated surfaces with respect to mimetic peptide surfaces (RGD and FHRRIKA) (34). Further *in vitro* cell adhesion assays should pre-treat cell suspensions with an LHRRVKI-containing peptide at various concentrations to determine the involvement of the heparin-binding domain in human cancer cell adhesion to BSP. Co-incubation of cells with an LHRRVKI-containing peptide and FN-derived GRGDSP peptide will provide additional information on the potential interplay between the two protein sequences in BSP-induced cancer cell adhesion.

A major limitation in the current study is the use of the alamarBlue^®^ assay to determine cell adherence. The assay is commonly used to quantify cytotoxicity and cell viability. Alternatively, adherent cells may be fixed and stained with crystal violet (CV) (69). Dying cells lose their ability to adhere. CV dye stains the DNA and proteins of cells (70). The number of attached cells can be counted by eluting the CV dye and reading the absorbance with a spectrophotometer (69). However, previous studies also solely used the alamarBlue^®^ assay to quantify cell adhesion (71–73). Furthermore, the alamarBlue^®^ assay was previously confirmed as a suitable method to measure the migration and invasion of choriocarcinoma cells (74). Therefore, we believe that using only the alamarBlue^®^ assay to evaluate cell adhesion did not negatively impact our results or conclusions.

The present results have important implications for drug development. Knockdown of BSP or the use of BSP inhibitors may be a more feasible and effective approach for cancer therapy than integrin-targeted therapeutics due to several reasons, that is, integrins sharing the same subunits and the complexity of the integrin signalling cascade. To date, only seven integrin inhibitors have been approved by the U.S. Food and Drug Administration. None of the current drugs on the market target the integrins αvβ3 or αvβ5 or are used in cancer therapy (75), emphasising the need for new cancer drugs to block integrin-ECM interactions and tumour progression. Previous studies on animal models showed that silencing of BSP in human breast cancer and human lung cancer cells inhibits the development of bone metastases (20,76,77), possibly owing to decreased expression levels of αvβ3 (77) and MMP-14 (20). Silencing of BSP may also suppress tissue remodelling by MMPs and inhibit cancer cell invasion into surrounding tissue, as intact BSP is able to stimulate the activity of proMMP-2 (51). *In vitro*, treatment of cancer cells with BSP enhances the binding of cancer cells to MMP-2 (53) and increases the mRNA expression of MMPs MMP-14, MMP-2 and MMP-9 (20,25), leading to enhanced cancer cell migration and invasion (20). Interfering with BSP expression may suppress the formation of skeletal metastases *in vivo*, in part, by inhibiting MMP-dependent cancer cell chemotaxis. Thus, future cancer drug development may focus on BSP-targeted therapeutics.

In conclusion, the present study demonstrated that the RGD sequence is essential to BSP-mediated adhesion of adenocarcinoma, prostate adenocarcinoma and NSCLC cells. Cancer cell attachment to BSP occurs through the binding of αvβ3 and αvβ5 integrins. The RGD-integrin interaction serves as a mechanistic link for the homing of cancer cells to bone. Targeting this cell-ECM adhesion with antibodies or RGD-containing peptides may provide a new approach to the prevention and treatment of skeletal metastases.

## Data Availability

The data generated in the present study may be requested from the corresponding author.

## Abbreviations

BSP: bone sialoprotein
ECM: extracellular matrix
GRGDSP: glycine-arginine-glycine-aspartic acid-serine-proline
RGD: arginine-glycine-aspartic acid
SIBLING: small integrin-binding ligand N-linked glycoprotein
